# Exploring the feasibility and acceptability of a sleep wearable headband among a community sample of chronic pain individuals: An at-home observational study

**DOI:** 10.1177/20552076221097504

**Published:** 2022-05-11

**Authors:** Zoe Zambelli, Cecilia E. Jakobsson, Laura Threadgold, Antonio R. Fidalgo, Elizabeth J. Halstead, Dagmara Dimitriou

**Affiliations:** 1 Sleep Education and Research Laboratory, Psychology and Human Development, UCL-Institute of Education, London, WC1H 0AA, UK; 2 School of Psychology, 4917 University of East London, London, E15 4LZ, UK

**Keywords:** Feasibility, acceptability, EEG, sleep quality, chronic pain

## Abstract

**Background:**

Chronic pain conditions affect up to one third of the adult population in the United Kingdom. Sleep problems are prevalent and negatively impact quality of life. Lack of standardised tools for routine screening and assessment of sleep changes have been a barrier for sleep management. Novel sleep wearables offer an exciting and accessible way to measure sleep but have not been tested outside of the consumer-led landscape and are not commonly used in research and clinical settings.

**Aims:**

The study aimed to explore the feasibility and acceptability of a sleep monitoring headband (Dreem 2) utilising EEG technology and accompanying smartphone application among a cohort of adults with chronic pain.

**Results:**

Twenty-one adults (81% women) completed a one-week home sleep study using a sleep headband and accompanying app. Ninety per cent of participants met the pre-defined requirement of two-night's sleep recording. All participants recorded one night of sleep data via the sleep headband. The majority (76%) of participants were satisfied with the sleep study, and 86% of participants were willing to wear the headband longer than the 2-night minimum requirement. Finally, 76% reported the headband as ‘somewhat’ or ‘extremely’ comfortable whist awake; 57% rated the headband as comfortable during sleep.

**Conclusion:**

The Dreem 2 headband appears to be a feasible and acceptable means of collecting sleep measurements among individuals with chronic pain, despite common sleep disturbances. These devices may have utility for screening, assessment and monitoring in research and practice. Further research is needed to provide guidelines and training for integration.

## Introduction

Non-cancer chronic pain affects up to one in three adults in the United Kingdom.^1^ Chronic pain has been associated with several health-related outcomes, such as physical co-morbidities, mental health, and quality of life outcomes, often in a bi-directional manner.^2–4^ In particular, sleep problems have been implicated as both a risk factor and exacerbating factor in developing and maintaining chronic pain.^5,6^ This population's most common sleep problems are insomnia-related disorders, often linked to initiating sleep and maintaining consolidated sleep.^7^

Although pain management aims to treat chronic pain within a biopsychosocial framework, evidence suggests that individuals with chronic pain often rely on pharmacological treatments, self-management and physical therapies as primary strategies to reduce the impact of pain and improve physical function.^8^ The routine care and management of chronic pain conditions seldom screens or targets sleep problems, despite the high prevalence of reported sleep problems in this population.^9,10^ In addition, research examining sleep architecture among individuals with chronic pain indicates deviations from healthy controls in the form of less rapid eye movement (REM) sleep, fewer sleep cycles, and a possible decrease in slow-wave-sleep (N3).^11–13^

Woo and Ratnayake^10^ concluded that the healthcare community recognised the detrimental impacts sleep problems have on health and wellbeing outcomes for individuals with chronic pain. However, due to a lack of standardised screening and protocols, it was challenging to identify the severity of the sleep issues and therefore refer for or conduct appropriate treatments. Another study conducted by Enam et al.^14^ identified a need for more training and education to promote the role of occupational therapy in addressing sleep management in nursing facilities. The authors also acknowledged the lack of standardisation in general screening, assessment and treatment of sleep problems.

The gold standard for sleep assessment is polysomnography (PSG), a procedure, which utilises electroencephalogram (EEG), electro-oculogram, electromyogram, electrocardiogram, pulse oximetry, in addition to respiratory effort, to measure underlying causes of sleep disturbances.^15^ Although effective at identifying sleep disorders and sleep parameters, PSG is an expensive sleep assessment. PSG may also be disruptive to patients as they must be assessed in a clinical setting. It is not routinely used to diagnose sleep disorders, with exception to sleep-related breathing disorders. Actigraphy refers to a commonly adopted method in research where an actigraph device is worn on the wrist to record movements that can estimate sleep parameters such as sleep onset latency, wake after sleep onset, and total sleep time.^16^ Despite its popularity through ease of implementation and cost-effectiveness, actigraphy cannot measure sleep architecture or changes therein, which may be a biomarker for chronic pain.^17–19^

The rapid increase in the development of health wearables has created a market for consumer sleep-tracking technology, which are easily accessible and often fitted with high-grade physiological sensors, including EEG, heart rate, temperature, and light exposure.^20^ These devices offer affordable means to sleep measurement beyond reliance on accelerometer data (actigraphy), whilst still enabling frequent and/or continuous data generation. Their potential unlocks an opportunity to analyse sleep metrics at a population level; however, their adoption within research and clinical practice is still limited. This may be due to the high number of devices which have come to market, and the inability of independent researchers to validate each device in a time-effective manner to lead to an uptake in practice, in addition to a lack of guidelines and protocols for settings outside of the consumer market itself.^20^ Further barriers potentially include the lack of expertise in interpreting these sleep parameters by groups of healthcare professionals, although many novel devices do aim to make interpretation and analysis of sleep tracking available to inexpert groups using automation and user friendly interfaces.^21^

Further to this, developers often conduct validation of their own products, which may provide a baseline for researchers to begin adopting these technologies. Given the link between chronic pain and sleep, the ability to measure sleep parameters through these technologies could benefit chronic pain patients and the healthcare providers treating them. A limited amount of literature has explored the feasibility and usability of these devices among clinical populations, an area that requires research and replicability to drive implementation in research and practice settings.^22^

In light of the above mentioned, the present study sought to explore the feasibility and acceptability of a wearable sleep-measuring device called Dreem 2 and address the following aims:

1. We sought to examine the feasibility of collecting data related to sleep utilising a sleep EEG headband, Dreem 2, within participants’ own homes, among adults with chronic pain conditions.
2. We assessed the acceptability and collected feedback on patient experience outcomes related to the wearability of the Dreem 2, in addition to the at-home implementation and induction of the device.

## Methods

### Design

The current feasibility study employed a cross-sectional design to evaluate whether Dreem 2 headbands are a practical research device for assessing sleep quality and quantity in adults who experience chronic pain in a home environment.^23^

### Procedure

#### Participants

Participants were recruited through an opt-in option from a previous research project at UCL.^24^ The Sleep Education and Research Laboratory created a webpage with information and a video recording about the study for those interested.

#### Screening

Participants registered their interest in the study via email and provided informed consent before partaking in the study. Participants completed a baseline questionnaire via the online platform Qualtrics, and eligible participants subsequently partook in a screening call via video conferencing which assessed against all inclusion and exclusion criteria. If participants failed to meet inclusion criteria, they were excluded at this stage, and baseline responses were not included in subsequent analyses (n = 2).

**Figure 1.**
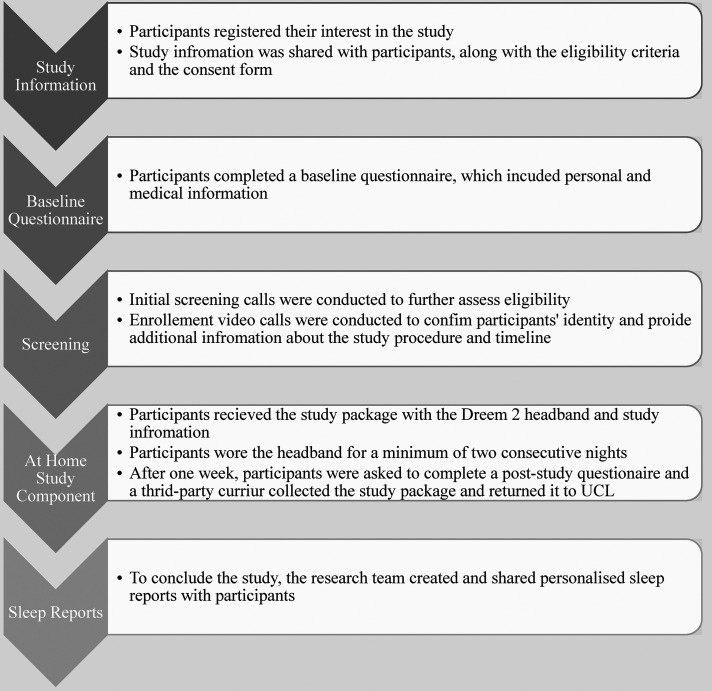
Study procedure *flow chart*.

Following assessment, participants were invited to an enrolment call via videoconferencing to clarify the study procedure and timeline further, ensure participants could download smartphone application and confirm the participants’ identity and address for the delivery of the study package. The researchers pre-assigned login details which were provided by the Dreem 2 developers for the purpose of this study. See Figure 1 for the study procedure.

#### Home study

Participants were sent the Dreem 2 headbands and were provided instructions on how to use the device. Participants were instructed to wear the Dreem 2 headband for a minimum of two consecutive nights; however, participants were in possession of the Dreem 2 headband for one week and were able to utilise them on as many nights as they wished to. Participants also completed a post-study questionnaire, which allowed them to provide feedback on the study and their experience using the Dreem 2 app and headband. Finally, to thank the participants, personalised sleep which included signposting and sleep hygiene recommendations were provided.

#### Eligibility criteria

Inclusion criteria were adults aged 18 years and above with a reported primary pain condition of chronic widespread pain or musculoskeletal pain. We further required that participants be able to speak, read, and understand English, live in the UK, and have access to a smartphone or tablet device for downloading the Dreem 2 application.

Exclusion criteria were factors that may interfere with sleep (e.g. psychopathology, substance abuse) or inability to complete the study (e.g. planned to stay away from home). Participants also did not meet inclusion criteria if they had a diagnosis of periodic limb movement disorder, sleep apnoea (participants were screened using the STOP-BANG questionnaire but not excluded on basis of screening alone), as well as a history of severe mental health requiring inpatient care.

#### Ethics

The study was granted ethical approval from UCL's Institute of Education Research Ethics Committee. The data protection reference for the study is Z6364106/2021/03/87. The study was funded by the Economic and Social Research Council.

#### Participant characteristics

Forty adults registered interest to partake in the study. Post-screening, 23 (57%) participants met the eligibility criteria. Two participants withdrew from the study during enrolment but prior to receiving their study package. A total of 21 participants took part in the home sleep study. Most participants identified as women (n = 17, 81.0%) and were of white ethnic origin (n = 20, 95.2%). All participants were diagnosed by a healthcare professional (HCP). Men reported chronic widespread pain as their primary condition, whereas women reported chronic widespread pain (n = 11, 64.7%), back pain (n = 1, 5.9%) and arthritis (n = 5, 29.4%). Most participants had experienced pain for between five and 10 years (n = 8, 28.1%) and all endorsed experiencing sleep problems for over one year. Please see Table 1 for participant characteristics.

**Table 1.** Participant characteristic*s*.

Variable	Male	Female	Total
*N* (%)	N = 4 (19.05)	N = 17 (80.95)	N = 21 (100.00)
**Age**	*M* = 43.25	*M* = 44.12	*M* *=* 43.95
	*SD* = 13.72	*SD* *=* 10.42	*SD* *=* 10.73
**Ethnicity**			
White	4 (100.00)	16 (94.12)	20 (95.24)
Black/ Black British	0 (0.00)	1 (5.88)	1 (4.76)
**Highest Level of Academic Attainment**			
Secondary school education or below	2 (50.00)	2 (11.76)	7 (33.33)
Higher education, undergraduate, postgraduate	2 (50.00)	15 (88.24)	15 (71.43)
**Employment**			
Full-time	3 (75.00)	6 (35.29)	9 (42.86)
Self-employed	1 (25.00)	4 (23.53)	5 (23.81)
Part-time		5 (29.41)	5 (23.81)
Unemployed		1 (5.88)	1 (4.76)
Home maker		1 (5.88)	1 (4.76)
**Primary Pain Condition**			
Chronic widespread pain/ fibromyalgia	4 (100.00)	11 (64.71)	15 (71.43)
Back pain		1 (5.88)	1 (4.76)
Arthritis/ Rheumatism		5 (29.41)	5 (23.81)
**Pain Duration**			
Less than 1 year	0 (0.00)	2 (11.76)	2 (9.52)
2-5 years	2 (50.00)	5 (29.41)	7 (33.33)
5-10 years	1 (25.00)	7 (41.18)	8 (38.10)
Over 10 years	1 (25.00)	3 (17.65)	4 (19.05)
**Diagnosis**			
HCP Diagnosed	4 (100.00)	17 (100.00)	21 (100.00)
**Pain Medication**			
Yes	2 (50.00)	15 (88.24)	17 (80.95)
No	2 (50.00)	2 (11.77)	4 (19.05)
**Sleep Problem Duration**			
Less than 1 Year	0 (0.00)	0 (0.00)	0 (0.00)
More than 1 Year	4 (100.00)	17 (100.00)	21 (100.00)

### Measures and materials

#### Dreem 2 headband

**Figure 2.**
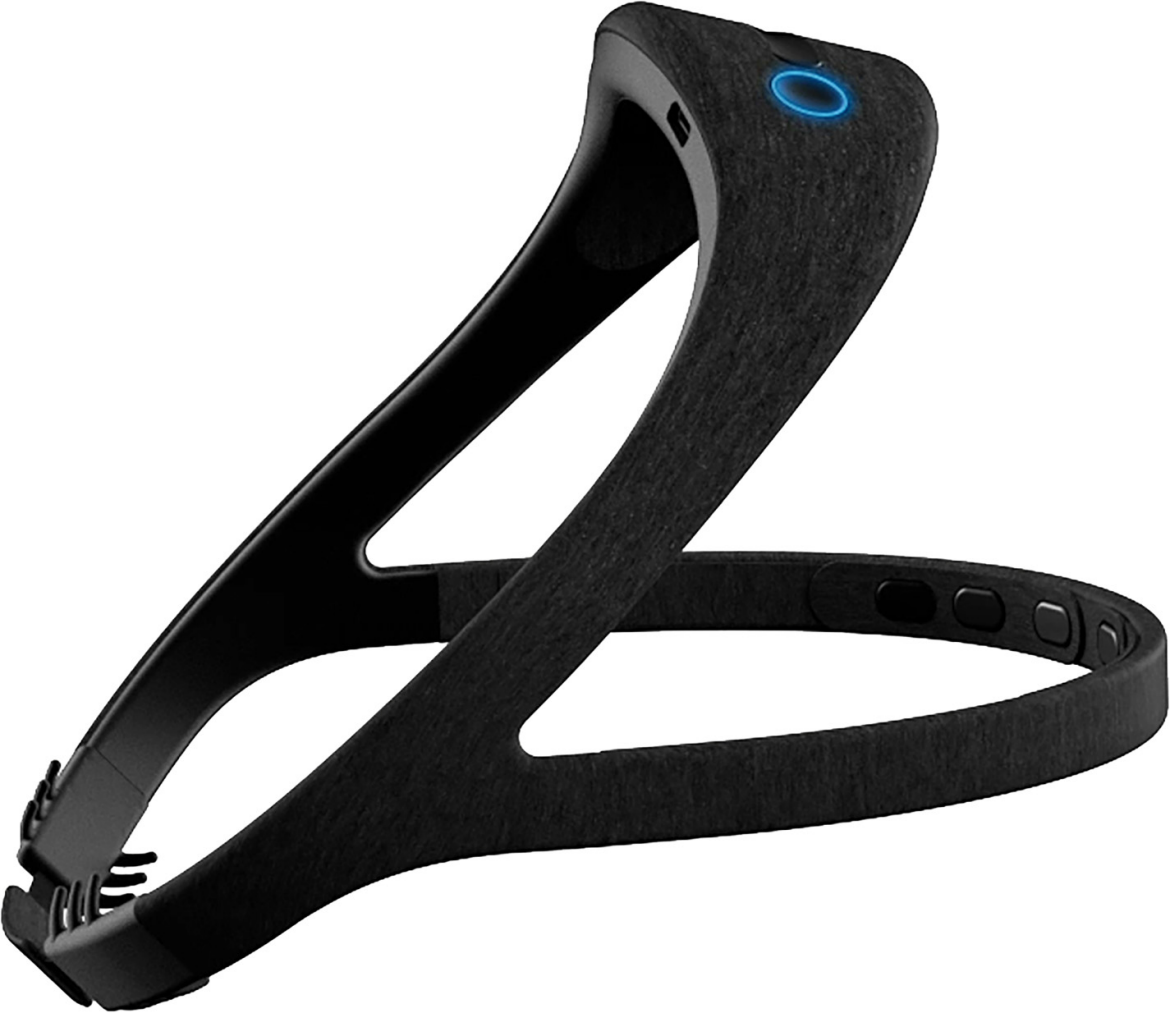
Dreem 2 headband.

The Dreem 2 headband is wireless and worn during sleep which records and stores physiological sleep data. The Dreem 2 headband contains three types of embedded sensors: (1) Five EEG dry electrodes yielding seven derivations to collect brain cortical activity data, (2) a 3D accelerometer located over the head which records movements, position, and breathing frequency, and (3) red-infrared pulse oximeter located in the frontal band which records heart rate.^23^ See Figure 2.

The Dreem 2 headband is used in conjunction with the ‘Dreem for research’ application. The app allows the transfer of stored Dreem 2 data onto a secure mobile application via Bluetooth. Users can subsequently view metrics regarding their night's sleep. Researchers can access data stored on the Dreem servers. The application can be accessed on both Android and Apple iOS and is free to download. For this study, participants were provided with app log-in details to ensure full data confidentiality and anonymity.

#### Demographics

Participants were asked to report their primary pain condition diagnosis and whether this was self-diagnosed or diagnosed via a healthcare professional (HCP). Within the baseline questionnaire, participants completed a self-reported health and medications assessment in which information regarding physical and mental health conditions was collected. Participants also provided information on their medication usage. Demographic variables included age, gender, ethnicity, educational attainment and employment status.

#### Baseline pain scores

Pain was assessed by a widely used self-report measure for clinical pain, namely the Brief Pain Inventory (BPI).^25^ The BPI is comprised of two subscales measuring (1) severity of pain and (2) the degree to which pain interferes with normal functioning over the past 24 h. Scores for each subscale range from 0 to 10, with higher scores indicating higher levels of pain severity and interference. Both subscales of the BPI have previously been validated in chronic pain populations.^26^ The BPI has shown good internal consistency across both subscales; α = 0.85.^27^

#### Baseline sleep quality scores

Sleep quality was assessed using the Pittsburgh Sleep Quality Index (PSQI).^28^ The PSQI is a validated instrument for sleep research containing a total of 24 items covering seven components. Components measure: (1) subjective sleep quality, (2) sleep latency, (3) sleep duration, (4) sleep efficiency, (5) sleep disturbance, (6) daytime dysfunction, and (7) sleep medication over the past month. Each component generates a score ranging from 0 to 3, with higher scores indicating poorer sleep quality outcomes. A global PSQI score is generated by summing scores across the seven components, with scores above five indicating poor sleep quality. The PSQI has previously been validated among chronic pain populations and exhibits good reliability (α = 0.7).^29^

#### Baseline anxiety and depression scores

The Hospital Anxiety and Depression Scale (HADS) was used to assess participants’ symptoms of anxiety and depression during the past week.^30^ This is a validated 14-item measure comprised of two subscales assessing anxiety (HADS-A) and depression (HADS-D). Participants respond to each item using four-point Likert scales ranging from 0 (not at all) to 3 (most of the time). Total HADS scores for each subscale are calculated by first reverse scoring five items and subsequently summing scores for each subscale. Higher HADS subscale scores indicate more severe symptoms, with scores above eight indicating anxiety and depression. The HADS has previously been validated in chronic pain populations, with demonstration of good internal reliability.^31^

#### Sleep diary

Paper sleep diaries were distributed to 50% (n = 13) of participants within the home study packs. These were included as a means to examine whether the added requirement of completing a sleep diary alongside using the Dreem 2 headband would impact the feasibility element of our study. It is also common for sleep diary data to be collected alongside objective sleep data to monitor lifestyle factors which may interfere with the sleep-wake cycle, although this was not within scope of our study.^32,33^ Participants were asked to complete the sleep diaries for five days within the home study week and days which coincided with use of the Dreem 2 headband. Daytime sleep diaries were filled in at the end of the day before participants went to bed whilst night-time sleep diaries were completed in the morning a short time after participants got out of bed.

The daytime sleep diary contained 17 self-report questions regarding factors within a participant's day. For example, participants provided information on their bedtime routine and any other factors influencing the typicality of their day. The night-time sleep diary contained 17 self-report questions regarding overnight factors. For example, participants were asked to recall information on the quality of their sleep including number of awakenings, disturbances, time in bed, time asleep and time taken to fall asleep.

#### Post-study acceptability questionnaire

A questionnaire was distributed to participants within the study pack and was completed on paper at the end of the study period. This contained 32 questions assessing experiences of using the Dreem 2 headband and app. For example, participants rated the ease of app and the extent to which the Dreem 2 headband interfered with their normal sleep pattern. The questionnaire was based on a previously used survey used in feasibility trial, adapted for the chronic pain population and study design.^34^ (See supplementary materials).

#### Analysis plan

Analyses were conducted using IBM SPSS v. 27. Descriptive analyses examined the means, standard deviations, and minimum and maximum values for each of the baseline measures. Exploratory analyses examined any significant differences in outcome measures based on gender, operating system type (e.g. Android vs. Apple iOS), and sleep diary group (completed vs. not completed).

Feasibility of the Dreem 2 headband was assessed by the overall rate of study completion across the sample as measured by the percentage of participants meeting the minimum 2-nights of successful recordings. Descriptive statistics regarding total number of complete recording nights and number of failed or incomplete recording attempts was also reported across the sample. Analysing the Dreem 2 headband data on the research server allowed comparisons between recordings and participants’ self-reports of their nights’ sleep within sleep diaries.

Acceptability of the Dreem 2 headband device was assessed by participants’ ratings of the comfort, wearability, and satisfaction of wearing the headband during sleep within the survey. Acceptability of the Dreem 2 app was measured by participants’ ratings of its user-experience, including ease of pairing, navigation, and troubleshooting.

## Results

### Baseline characteristics

Table 2 displays the mean scores for all baseline measures. The mean PSQI global score across the sample were observed to be above the threshold (>5) for poor sleep quality. Furthermore, the average sleep efficiency across the sample was reported as 68.1%, significantly below the optimal range of between 85% and 90%.^35^ The mean Epworth score across the sample was shown to fall within the ‘normal’ range for sleepiness, with almost a full range of scores observed. The mean HADS-A (anxiety) and HADS-D (depression) scores fell within the ‘normal’ range for symptoms of anxiety and depression among the sample.

**Table 2.** Mean, standard deviation, minimum, and maximum scores for baseline measure characteristic*s*.

Sleep Scale	Individual Items	Mean	SD	Minimum	Maximum
Pittsburgh Sleep Quality Index	Minutes to Fall Asleep	1.52	1.12	0.00	3.00
	Sleep Latency	2.05	0.92	1.00	3.00
	Sleep Duration	1.29	1.01	0.00	3.00
	Time in bed (hrs)	8.78	1.34	6.25	11.5
	Sleep efficiency (%)	68.14	16.13	28.57	97.30
	PSQI Sleep Efficiency	1.85	1.09	0.00	3.00
	Sleep Disturbance	2.05	0.50	1.00	3.00
	Sleep Medication	0.95	1.36	0.00	3.00
	Daytime dysfunction	1.52	0.81	0.00	3.00
	PSQI Global Score	11.43	3.67	4.00	17.00
Brief Pain Inventory	BPI Severity	4.53	1.61	2.00	7.75
	BPI Interference	5.47	1.61	1.86	7.75
Hospital Anxiety and Depression Scale	HADS Anxiety	7.38	4.09	1.00	14.00
	HADS Depression	7.43	3.31	2.00	13.00

Outcome measure score ranges with higher scores indicating higher severity of symptoms: PSQI global 0-21 points, BPI: 0-10 points, HADS: 0-21 points.

### Sleep parameters recorded via Dreem 2 headband

Table 3 displays the main sleep parameters recorded by the Dreem 2 headband during the home sleep studies. On average, participants recorded a mean of 24 min sleep onset latency, and just over 31 min of sleep after wake onset. Total sleep time was recorded, on average, as 428 min (7.13 h) across the group, and sleep efficiency (SE) was close to 85%. Finally, the average time spent in non-REM sleep (i.e. stage 1,2, and slow wave sleep) was 75% of the night recordings, across the group.

**Table 3.** Mean, standard deviation, minimum, and maximum scores for sleep parameters recorded via Dreem 2.

Dreem 2 variable	M (minutes/%)	SD (minutes/%)	Min (minutes/%)	Max (minutes/%)
Sleep onset latency	24.52	21.21	5	102
**Wake after sleep onset**	31.33	19.64	8	89
**Sleep duration**	427.67	57.12	267	502
**Sleep efficiency (%)**	84.81	6.12	72	94
**REM sleep (%)**	24.57	4.92	15	35
**Non- REM sleep (%)**	75.43	4.92	65	85

### Feasibility of the Dreem 2 headband

Overall, frequency analyses revealed 95.2% of participants met the study requirement of a minimum of 2-night complete Dreem 2 headband recordings. All participants successfully completed one night of sleep recording. The mean number of complete recordings was 4 nights’ recordings (see Table 4 for details). The mean number of self-reported nights wearing the Dreem 2 headband was similar: M = 4.62 nights’ recordings. There were 16 incidences of incomplete recordings across the whole sample (Table 4). On average, participants reported removing the Dreem 2 headband device 1.38 times throughout the home study period.

**Table 4.** Self-reported and objected Dreem 2 headband data across operating systems and sleep diaries and whole sample.

Comparison group	Descriptive statistics	No. nights with complete sleep recordings	No. nights with incomplete sleep recording	No. nights participant attempted to use the headband	No. nights participant wore headband	No. nights participant removed the headband during the night
iOS	Mean	4.00	0.50	5.00	4.25	1.88
	SD	1.69	0.93	1.41	1.75	1.36
	Minimum	2.00	0.00	3.00	2.00	1.00
	Maximum	6.00	2.00	6.00	6.00	5.00
ANDROID	Mean	4.46	0.38	4.92	4.85	1.08
	SD	2.18	0.87	1.19	1.35	0.28
	Minimum	1.00	0.00	3.00	2.00	1.00
	Maximum	9.00	3.00	6.00	6.00	2.00
No sleep diary	Mean	5.00	0.33	5.50	5.50	1.17
	SD	1.26	0.82	0.55	0.55	0.41
	Minimum	4.00	0.00	5.00	5.00	1.00
	Maximum	7.00	2.00	6.00	6.00	2.00
Sleep diary completed	Mean	4.00	0.47	4.73	4.27	1.47
	SD	2.17	0.92	1.39	1.62	1.06
	Minimum	1.00	0.00	3.00	2.00	1.00
	Maximum	9.00	3.00	6.00	6.00	5.00
Whole sample	Mean	**4** **.** **29**	**0**.**43**	**4**.**95**	**4**.**62**	**1**.**38**
	SD	1.98	0.87	1.24	1.50	0.92
	Minimum	1.00	0.00	3.00	2.00	1.00
	Maximum	9.00	3.00	6.00	6.00	5.00

### Operating system and sleep-diary group differences in feasibility

Independent samples t-tests revealed no significant difference in the number of complete recordings based on operating system or sleep-diary group (p > .05). Furthermore, there were no significant differences in the number of incomplete recordings across groups. Participants’ self-reports of using the Dreem 2 headband attempts, removal and use did not significantly differ based on these groupings (Table 4).

### Acceptability of the Dreem 2 headband and app

The majority (81%) of participants rated the overall ease of the app as extremely or somewhat easy to use throughout the study. Furthermore 91% found downloading the app for the device to be extremely easy. However, when rating ease of pairing the Dreem 2 headband with the app, only half (48%) of participants reported the experience as ‘extremely’ easy. Indeed, 43% of participants reported having to attempt pairing the device with the app more than once. Furthermore, almost half (43%) of participants reported receiving no feedback from the app across the study (see below disclaimer relating to an identified issue).

A bug was identified within the iOS app shortly after commencement of the first cohort study which revealed a pairing issue among iOS users. In addition, there was no sleep feedback available to these participants. Although this did not impact the recording and uploading of the raw data to the researcher's online server, the issue was communicated to the development team who were able to fix this before the second cohort. Subsequent cohorts did not experience any issues related to the above.

The majority (76%) of participants were satisfied with the overall sleep study and what it entailed. In addition, 86% of participants reported willingness to wear the Dreem 2 headband longer than the 2-night minimum requirement. Regarding comfort, most participants (76%) reported the Dreem 2 headband ‘somewhat’ or ‘extremely’ comfortable whist awake. Over half (57%) of participants rated the Dreem 2 headband as comfortable during sleep, with almost one third of participants rating the Dreem 2 headband as ‘somewhat’ uncomfortable (see supplementary material).

### Operating system and sleep-diary group differences in acceptability

Descriptive analyses showed differences in ratings of the app based on whether participants had Android or iOS operating systems (Table 5). However, independent samples t-test revealed no statistical difference between operating system groups (p > 0.05). Participants with Android devices (76.9%) were more likely to rate app instructions as extremely good compared to those with iOS devices (25.0%). Moreover, the majority (61.5%) of Android participants rated the overall ease of the app during the study as extremely good. In comparison, a minority (25.0%) of participants with iOS operating devices did so. Despite this, there were no large differences in overall satisfaction with the study based on the operating system group (Table 5).

**Table 5.** Differences in self-report ratings of the App based on operating system group.

Measure	Operating System	
	Android *n* = 13 (56.52)	iOS *n* = 8 (34.78)
**Rating of app instructions [*n* (%)]**		
Extremely good	10 (76.92)	2 (25.00)
Somewhat good	1 (7.69)	5 (62.50)
Neither good nor bad	2 (15.38)	1 (12.50)
**Overall ease of using app during sleep study [*n* (%)]**		
Extremely easy	8 (61.54)	2 (25.00)
Somewhat easy	3 (23.08)	4 (50.00)
Neither easy nor difficult	1 (7.69)	0 (0.00)
Somewhat difficult	1 (7.69)	2 (25.00)
**Satisfaction with sleep study based on headband [*n* (%)]**		
Extremely satisfied	6 (46.15)	3 (37.50)
Somewhat satisfied	3 (23.08)	4 (50.00)
Neither satisfied nor dissatisfied	4 (30.77)	0 (0.00)
Somewhat dissatisfied	0 (0.00)	0 (0.00)
Extremely dissatisfied	0 (0.00)	1 (12.50)

Descriptive analyses of participants’ self-report post-study questionnaire feedback revealed some differences in ratings of the Dreem 2 headband based on sleep diary group; however, similarly the independent samples t-test revealed no statistical difference between sleep diary groups (p > 0.05) (Table 6). The majority (66.7%) of participants asked to complete a sleep diary throughout the study reported that the Dreem 2 headband disturbed their sleep to some extent. In comparison, the majority (66.7%) of participants who were not asked to complete a sleep diary reported that the Dreem 2 headband did not disturb their sleep at all (Table 6). Furthermore, participants in the sleep diary group (60.0%) were more likely to be extremely or somewhat satisfied with the sleep study compared to those in the no sleep diary group (100.0%).

**Table 6.** Differences in self-report ratings of Dreem 2 headband study based on sleep diary group*.*

Measure	Operating System	
	Sleep Diary *n* = 15 (65.22)	No Sleep Diary *n* = 6 (24.08)
**Extent to which headband disturbed sleep [*n* (%)]**		
A great deal	1 (6.67)	0 (0.00)
A moderate amount	4 (26.67)	1 (16.67)
A little	5 (33.33)	1 (16.67)
None at all	5 (33.33)	4 (66.67)
**Satisfaction with sleep study based on headband [*n* (%)]**		
Extremely satisfied	7 (46.67)	2 (33.33)
Somewhat satisfied	2 (13.33)	4 (66.67)
Neither satisfied nor dissatisfied	4 (26.67)	0 (0.00)
Somewhat dissatisfied	0 (0.00)	0 (0.00)
Extremely dissatisfied	1 (6.67)	0 (0.00)

### Compliance of the Dreem 2 headband and App

The study had high compliance, no participant dropped out and all participants attempted to wear the Dreem 2 headband for the recommended minimum 2 nights. A rudimentary thematic analysis of written responses revealed there were barriers to utilising the Dreem 2 headband. Two participants found pairing the device before each use difficult and explained that it was frustrating if the data took a long time to transfer in the morning. Participants with a better connection enjoyed viewing their sleep data on the app each morning.

Suggestions for how the device could improve were made, these included: (1) receiving confirmation via the app when the data were successfully downloaded; (2) improved adjustable straps; and (3) a more straightforward means to pairing. Overall, participant's satisfaction with the Dreem 2 headband appeared linked to whether they had any technical or practical difficulties using the device

Several participants claimed that despite the adjustable straps offered in three sizes, the Dreem 2 headband would become loose overnight, and the connection would be disrupted with movement.

## Discussion

This study aimed to explore the feasibility and acceptability of a sleep monitoring Dreem 2 headband device among a community sample of individuals with chronic pain. Overall, we learned that the use of the device was feasible among the group. Over 95% of participants were able to use the device to record sleep data for two nights during an allocated study period. In addition, all participants provided recording from at least one night of sleep data. The study uncovered potential barriers to the use of the Dreem 2 headband, in particular, highlighting the need for clear guidance on pairing the headband with a personal device via Bluetooth and general troubleshooting for when technical issues may arise. In addition, most participants were satisfied with the home study and indicated a willingness to wear the Dreem 2 headband for a period longer than two nights.

The findings from this study have implications for the use of sleep monitoring devices among chronic pain communities. Firstly, due to the lack of routine and standardised assessment of sleep in this population,^10^ EEG headbands such as a Dreem 2 headband offer a comprehensive view of micro and macro sleep architecture. Given that chronic pain populations have been shown to experience reduced slow wave sleep and REM sleep compared to healthy controls,^36^ monitoring sleep using EEG would allow researchers and/or clinicians to better understand the long-term development of sleep architecture, in response to medication and other treatments which are offered for pain management. For example, the use of opioid medications, among other analgesic drugs, are known to reduce REM and SWS.^37,38^ Implementing devices which allow for easy monitoring of sleep changes at initiation of new pain management could improve overall outcomes and allow a more tailored approach to treatment at individual patient level.

The study also highlights a few barriers and facilitators to uptake. Firstly, participants must have digital literacy to use the device independently, in particular where the aim is to monitor in a home environment. As such, this excludes individuals without a stable internet connection and a suitable smart device, barriers which are known to cause further health inequalities in digital health uptake.^39^ In addition, we found the Dreem app's functionality to present basic sleep metrics back to the participant encouraged use of the Dreem 2 headband and was a likely contributor to adherence and compliance in our feasibility study. This is in line with Ferguson et al.^40^ who found a key facilitator to adherence of wearable technology among cardiac patients were ‘timely and appropriate feedback, and friendly user experience’. Although promising, it is important to recognise that the feasibility and acceptability of the Dreem 2 headband is one aspect of integrating these technologies into practice; validating the headband data, funding for devices, and training development for researchers and clinicians are equally important in facilitating the uptake of these devices.^41,42^ Despite limited literature on feasibility among clinical populations of these sleep monitoring devices, our findings mirror those of Dunn et al.^22^ which demonstrated high compliance rates (87%) among a cohort of individuals with Opioid Use Disorder (OUD) trialling a similar sleep monitoring device. Similar results have been found in healthy young adults.^34^

### Implications and future directions

1. A Dreem 2 headband may be a feasible and acceptable device to collect comprehensive sleep data among individuals with chronic pain conditions
2. Our findings suggest provision of sleep feedback to participants within the user interface of the Dreem application may facilitate adherence and should be considered in future protocols
3. Full integration of these novel sleep technologies likely requires (1) training development for researchers and clinicians in addition to (2) further evidence of validity against gold-standard sleep measurement
4. An economic analysis highlighting cost-effectiveness of these novel sleep technologies will increase their potential to become a standardise tool in pain management, where sleep issues are known to impact quality of life which is one direction of future research is needed to increase uptake

### Limitations

Firstly, this study included a community-dwelling sample of individuals with chronic pain and is therefore not representative of perhaps most severe cases. Secondly, the sample was limited to individuals with musculoskeletal conditions, other chronic pain conditions such a chronic headache were excluded from this study due to limited number of devices available. Future feasibility studies should expand the participant pool to include other conditions. Third, the majority of the sample was from higher educational background which may have contributed to the feasibility results, given that digital literacy was identified as a facilitator in this regard. Furthermore, links between lower socioeconomic status and digital health literacy have been studied.^43,44^ Finally, the study did not assess the validity of the sleep scoring algorithm which provides the sleep data at individual level. One validation study has reported good congruence between the automated algorithm and human-scored sleep data from the Dreem 2 device.^23^

## Conclusion

The link between sleep and chronic pain has been demonstrated in several research studies, as such, there is agreement that targeting sleep problems is important to improving health-related outcomes in this population.^45–47^ Despite a drive toward a holistic care model in treating chronic pain, barriers to adequately assessing and screening sleep problems may be one reason that targeted treatments are not offered routinely to this population. The advancement of technology has witnessed the emergence of several novel sleep tracking devices arrive to the consumer market, ranging from accelerometer-based technology to the incorporation of EEG and heart sensors. Our study offers one of the first reporting feasibility and acceptability of the Dreem 2 headband among chronic pain populations which we hope will inform their use in practice or research settings.

## Supplemental Material

sj-docx-1-dhj-10.1177_20552076221097504 - Supplemental material for Exploring the feasibility and acceptability of a sleep wearable headband among a community sample of chronic pain individuals: An at-home observational study Click here for additional data file. Supplemental material, sj-docx-1-dhj-10.1177_20552076221097504 for Exploring the feasibility and acceptability of a sleep wearable headband among a community sample of chronic pain individuals: An at-home observational study by Zoe Zambelli, Cecilia E. Jakobsson, Laura Threadgold, Antonio R. Fidalgo, Elizabeth J. Halstead and Dagmara Dimitriou in Digital Health

sj-docx-2-dhj-10.1177_20552076221097504 - Supplemental material for Exploring the feasibility and acceptability of a sleep wearable headband among a community sample of chronic pain individuals: An at-home observational study Click here for additional data file. Supplemental material, sj-docx-2-dhj-10.1177_20552076221097504 for Exploring the feasibility and acceptability of a sleep wearable headband among a community sample of chronic pain individuals: An at-home observational study by Zoe Zambelli, Cecilia E. Jakobsson, Laura Threadgold, Antonio R. Fidalgo, Elizabeth J. Halstead and Dagmara Dimitriou in Digital Health
