# Supplementary material for: Exploring the feasibility and acceptability of a sleep wearable headband among a community sample of chronic pain individuals: An at-home observational study
Source: Digit Health. 2022 May 11;8:20552076221097504. doi: 10.1177/20552076221097504 (PMC9102155; doi:10.1177/20552076221097504)
Supplement: sj-docx-1-dhj-10.1177_20552076221097504 - Supplemental material for Exploring the feasibility and acceptability of a sleep wearable headband among a community sample of chronic pain individuals: An at-home observational study [file sj-docx-1-dhj-10.1177_20552076221097504.docx]

UCL Home Sleep Study Post-study Survey

Thank you for completing our Home Sleep Study, we hope you enjoyed the experience and that we can use the learning from this to improve future sleep health among chronic pain individuals. Please complete this short survey by answering some questions about your experience of using the DREEM headband and how you found the experience of taking part in the study overall.

You should complete this survey toward the end of your study allocation- **please don't forget to send it back with rest of the study package!**

Please write your first name

________________________________________________________________

Please write your surname

________________________________________________________________

Q1 Please rate the ease of downloading the DREEM app to your smartphone or tablet

- Extremely easy
- Somewhat easy
- Neither easy nor difficult
- Somewhat difficult
- Extremely difficult

Q2 Please rate the pairing process for the DREEM app and DREEM headband i.e. connecting your DREEM headband to Wi-Fi

- Extremely easy
- Somewhat easy
- Neither easy nor difficult
- Somewhat difficult
- Extremely difficult

Q3 How many attempts did it take to pair the DREEM headband with the DREEM app and WiFi?

- 1 attempt (it worked the first time)
- 2 attempts
- 3 or more attempts

Q4 Please rate the DREEM app instructions to help start a night recording

- Extremely good
- Somewhat good
- Neither good nor bad
- Somewhat bad
- Extremely bad

Q25 Did you receive feedback on your night's sleep via the DREEM app during your sleep study?

- Yes
- No
- Unsure

Q26 If yes, how useful or of interest to you was the feedback via the DREEM app regarding your sleep?

- Extremely useful
- Very useful
- Moderately useful
- Slightly useful
- Not at all useful

Q5 Overall, please rate the ease of using the DREEM app during your home sleep study

- Extremely easy
- Somewhat easy
- Neither easy nor difficult
- Somewhat difficult
- Extremely difficult

Q6 On how many nights did you attempt to use the headband to record your sleep? i.e. you followed the instructions through to adjust the headband onto your head before bedtime.

- 0 nights
- 1 night
- 2 nights
- 3 nights
- 4 nights
- 5 or more night
- Unsure/ Can't remember

Q7 On how many nights, were you able to keep the headband on throughout the night? i.e. kept the device on until morning/ wake time.

- 0 nights
- 1 night
- 2 nights
- 3 nights
- 4 nights
- 5 or more nights
- Unsure/ Can't remember

Q11 On how many nights did you remove the headband during the night? E.g. the DREEM device became misplaced or was removed out of discomfort.

- 0 nights
- 1 night
- 2 nights
- 3 nights
- 4 nights
- 5 or more nights
- Unsure/ Can't remember

Q21 Would you be willing to wear the headband for longer than the study period (two nights)?

- No
- Yes, 1 more night
- Yes, 2 more nights or longer

Q12 Please rate the ease of charging the headband

- Extremely easy
- Somewhat easy
- Neither easy nor difficult
- Somewhat difficult
- Extremely difficult

Q14 Please rate the comfort of the headband while AWAKE (e.g., before going to sleep)

- Extremely comfortable
- Somewhat comfortable
- Neither comfortable nor uncomfortable
- Somewhat uncomfortable
- Extremely uncomfortable

Q15 Please rate the comfort of the headband while attempting to SLEEP

- Extremely comfortable
- Somewhat comfortable
- Neither comfortable nor uncomfortable
- Somewhat uncomfortable
- Extremely uncomfortable

Q16 Please rate how much the headband disturbed your sleep, if at all

- A great deal
- A lot
- A moderate amount
- A little
- None at all

Q17 If the headband disturbed your sleep, please let us know how: (Indicate all that apply)

- I had more difficulty falling asleep than usual
- I woke up more frequently than usual
- I woke up earlier than usual and could not fall back asleep
- I felt more tired in the morning than usual
- Other, please specify ________________________________________________

Q18 Please indicate your typical preferred sleeping position: (select all that apply)

- Lying on my back
- Lying on my front (stomach)
- Lying on my side
- I switch positions frequently through the night
- All positions are equally preferred

Q19 Did the headband cause you to sleep in a different position than usual?

- Yes
- No
- Unsure

Q20 Please rate your satisfaction with the sleep study based on your experience with the headband

- Extremely satisfied
- Somewhat satisfied
- Neither satisfied nor dissatisfied
- Somewhat dissatisfied
- Extremely dissatisfied

Q21 Would you recommend the sleep study to a friend or family member based on your experience with the headband?

- Definitely yes
- Probably yes
- Might or might not
- Probably not
- Definitely not

Q22 If you have a bed partner, were they impacted by the headband at all?

- Yes
- No
- Unsure

Q23 If yes, please briefly explain how you bed partner was impacted by the DREEM device.

________________________________________________________________

Q27 Do you have any other comments regarding the home sleep study?

Thank you for once again for completing the study. Once your headband has been collected from your home and sent back to UCL, we will analyse the data and we will send you a **personalised sleep report** based on your recordings. If you have any questions or comments about the study and your experience you would like to share with the research team, please get in touch with XXXX
