# Supplementary material for: Exploring the feasibility and acceptability of a sleep wearable headband among a community sample of chronic pain individuals: An at-home observational study
Source: Digit Health. 2022 May 11;8:20552076221097504. doi: 10.1177/20552076221097504 (PMC9102155; doi:10.1177/20552076221097504)
Supplement: sj-docx-2-dhj-10.1177_20552076221097504 - Supplemental material for Exploring the feasibility and acceptability of a sleep wearable headband among a community sample of chronic pain individuals: An at-home observational study [file sj-docx-2-dhj-10.1177_20552076221097504.docx]

**Supplementary Table**

*Post-study questionnaire responses across whole sample analysing acceptability of the Dreem 2 headband device and app*

| **Acceptability Measure** | **No. Response Endorsed** |
| --- | --- |
| **Ease of using app during sleep study [*n* (%)]**  Extremely easy  Somewhat easy  Neither easy nor difficult  Somewhat difficult  Extremely difficult | 10 (47.62)  7 (33.33)  1 (4.76)  3 (14.29)  0 (0.00) |
| **Comfort of headband while awake [*n* (%)]**  Extremely comfortable  Somewhat comfortable  Neither uncomfortable nor uncomfortable  Somewhat uncomfortable  Extremely difficult | 6 (28.57)  10 (47.62)  3 (14.29)  2 (9.52)  0 (0.00) |
| **Comfort of headband whilst attempting to sleep [*n* (%)]**  Extremely comfortable  Somewhat comfortable  Neither uncomfortable nor uncomfortable  Somewhat uncomfortable  Extremely uncomfortable | 4 (19.05)  8 (38.10)  3 (14.29)  6 (28.57)  0 (0.00) |
| **Extent to which headband disturbed sleep [*n* (%)]**  A great deal  A moderate amount  A little  None at all | 1 (4.76)  5 (23.81)  6 (28.57)  9 (42.86) |
| **No. of nights headband removed due to disturbance or misplacement*** | 1.38 (0.92) |
| **Headband Cause to Sleep in Different Position [*n* (%)]**  Yes  No  Unsure | 2 (9.52)  15 (71.43)  4 (19.05) |
| **Willingness to wear headband longer than study requirements [*n* (%)]**  No  Yes, 1 more night  Yes, 2 more nights or longer | 3 (14.29)  3 (14.29)  15 (71.43) |
| **Satisfaction with sleep study based on headband [*n* (%)]**  Extremely satisfied  Somewhat satisfied  Neither satisfied nor dissatisfied  Somewhat dissatisfied  Extremely dissatisfied | 9 (42.86)  7 (33.33)  4 (19.05)  0 (0.00)  1 (4.76) |

*Values represent Mean (Standard Deviation)
